# Accumulation of Heavy Metals in Blueberry Floral Rewards and Their Effects on Reproductive Fitness and Bumblebee Pollination Behavior

**DOI:** 10.3390/plants15111656

**Published:** 2026-05-28

**Authors:** Lei Wu, Qi Sun, Xing Wang, Duo Liu, Yanwen Zhang

**Affiliations:** 1 School of Life Sciences, Changchun Normal University, Changchun 130032, China; wul1016@126.com (L.W.); sunqi01@ccsfu.edu.cn (Q.S.); 2 School of Agriculture, Liaodong University, Dandong 180003, China; wangxing529@126.com

**Keywords:** heavy metals, floral rewards, bumblebee, pollination behavior, reproductive fitness

## Abstract

Heavy metals in soil can be translocated to floral rewards via specific physiological pathways, potentially altering pollinator behavior and reducing plant reproductive fitness. However, the comparative effects of different heavy metals on floral rewards and pollination remain poorly understood. Here, blueberries were used as a model system to examine the accumulation of four heavy metals (Zn, Pb, Cu, and Ni) in floral rewards and assess their impacts on plant reproductive performance and bumblebee visitation behavior under controlled conditions. Heavy metals accumulated in floral rewards in a concentration-dependent manner, with strong positive relation to soil metal levels; pollen generally contained higher concentrations than nectar. Increasing soil heavy metal concentrations significantly shortened bumblebee visit duration to individual flowers, with the greatest reduction under high Pb treatment (26.32%), followed by Zn (21.49%), Ni (17.70%), and Cu (15.40%). Visit frequency also declined under medium to high concentrations. All heavy metal treatments reduced pollen viability (1.62–24.48%), pollen deposition on stigmas (9.91–40.59%), and single-fruit weight (16.25–32.51%), with effect magnitudes varying by metal (generally Pb > Cu > Zn > Ni). Overall, heavy metal accumulation in floral rewards negatively affects pollinator behavior and compromises plant reproductive fitness, with metal-specific effect strengths.

## 1. Introduction

Heavy metals represent a class of chemically toxic elements that pose significant risks to both the environment and living organisms. These elements can infiltrate plants via contaminated soil and water sources, as well as through floral rewards [1]. When floral rewards, such as nectar and pollen, accumulate heavy metals to certain concentrations, their physicochemical properties are altered, which in turn affects insect pollination behavior and plant reproductive fitness [2,3]. For example, bumblebees pollinating sunflowers cultivated in soil with elevated cadmium (Cd) concentrations demonstrated a marked decrease in visitation frequency and single-flower visit duration [4]. Heavy metals, including cadmium (Cd), copper (Cu), and lead (Pb), significantly diminished both the number of visits and the foraging duration for honeybees and bumblebees [5]. Furthermore, honeybees that ingested aluminum-containing nectar displayed detrimental effects on learning, orientation, and foraging behaviors [6]. Bumblebee foraging duration is influenced by the nickel (Ni) content in nectar, although aluminum does not appear to have a similar effect [7]. Exposure to heavy metals such as lead (Pb), copper (Cu), and arsenic has been shown to negatively affect cognitive function, learning, and brain development in bees [8,9,10]. Given the intrinsic properties of heavy metals, various species and concentrations exert differing effects on insect behavior through complex mechanisms, and research examples in this domain remain limited.

With respect to reproductive fitness, elevated concentrations of heavy metals such as lead (Pb), nickel (Ni), zinc (Zn), and copper (Cu) have been shown to substantially impair pollen performance. Specifically, pollen germination rates in *Pyrus pyrifolia*, *Armeniaca vulgaris*, and *Arabis alpina* were reduced by 21% to 98%, accompanied by a shortening of pollen tube length by 56.1 to 156 μm [11,12,13,14]. Additionally, chromium (Cr) inhibited seed germination of *Medicago sativa* by up to approximately 54% [15], while *Carthamus tinctorius* seeds completely failed to germinate under high cadmium (Cd) stress [16]. Our previous studies further demonstrated that exposure to Pb and Ni resulted in a reduction of approximately 10 mg in single-seed weight of *Cucurbita pepo*, while simultaneously causing a significant decline in pollen viability, thereby reducing male reproductive fitness [17]. In contrast, another study reported that within a certain concentration range, heavy metal accumulation in floral organs enhanced fruit set and seed germination in *Hosta ensata*, leading to increased female reproductive fitness [18]. Collectively, these findings suggest that the effects of heavy metals on plant reproductive fitness are highly complex and context-dependent, with outcomes varying across plant species, metal types, and exposure concentrations. Nevertheless, empirical evidence addressing these contrasting effects remains limited.

This study investigates the effects of nitrate solutions containing four heavy metals (Zn, Pb, Cu, and Ni) on blueberry growth under greenhouse conditions, specifically focusing on the capacity of blueberry floral rewards to accumulate these heavy metals. Additionally, the research examines how such accumulation impacts the foraging behavior of bee pollinators and the reproductive fitness of blueberries. The study aims to address the following questions: (1) To what extent do heavy metals applied to the substrate accumulate in blueberry floral rewards? (2) How does heavy metal accumulation in floral rewards influence pollinator visitation behavior? (3) In what ways does heavy metal accumulation affect male and female reproductive fitness in blueberries? Furthermore, elucidating the differential effects of various heavy metals is expected to provide a scientific foundation for developing proactive management strategies aimed at mitigating challenges associated with crop production in heavy-metal-contaminated environments.

## 2. Results

### 2.1. Heavy Metal Accumulation in Floral Rewards

**Figure 1:**
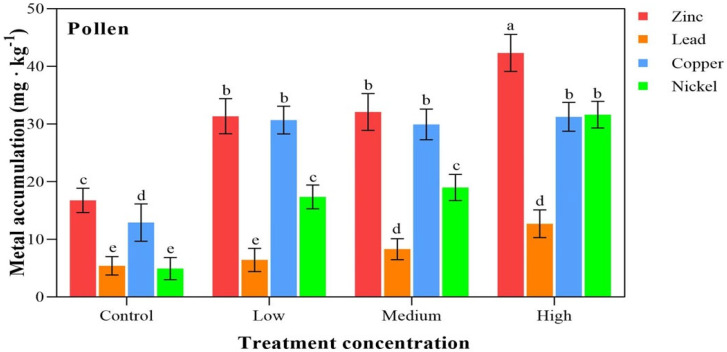
Content of heavy metals in blueberry flower pollen. Values are presented as mean ± SE. Different letters within each metal indicate statistically significant differences among groups (*p* < 0.05).

**Figure 2:**
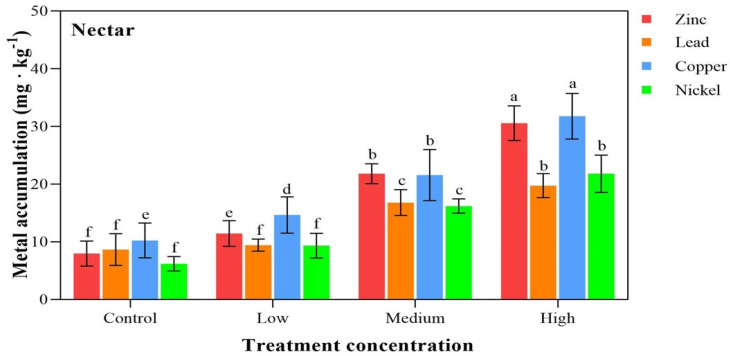
Content of heavy metals in blueberry flower nectar. Values are presented as mean ± SE. Different letters within each metal indicate statistically significant differences among groups (*p* < 0.05).

The content of heavy metals in floral rewards (pollen and nectar) following soil heavy metal application is presented in Figure 1 and Figure 2. Analysis of variance (ANOVA) results are presented in Table 1 and Table 2. Concentrations of heavy metals in blueberry floral rewards increased significantly with increasing soil contamination levels, indicating a clear positive relationship between soil-applied and floral heavy metal concentrations. Across treatments, pollen consistently exhibited higher heavy metal concentrations than nectar, particularly for Zn and Ni. In pollen, Zn and Cu displayed similar accumulation patterns, with concentrations significantly exceeding those of the control under low, medium, and high treatments (*p* < 0.05). The highest Zn concentration reached 42.33 ± 3.21 mg·kg^−1^, while Cu peaked at 31.27 ± 2.50 mg·kg^−1^. Nectar showed comparable trends, with Cu reaching a maximum concentration of 31.77 ± 3.95 mg·kg^−1^, which did not differ significantly from Zn content (30.57 ± 3.00 mg·kg^−1^; *p* > 0.05). At high exposure levels, no significant difference was observed between Cu and Ni content in pollen, whereas Cu accumulation in nectar was significantly higher than that of Ni. Overall, under high-concentration treatments, the content of individual heavy metals in both pollen and nectar was significantly greater than in the control group (*p* < 0.05). At equivalent soil contamination levels, distinct content patterns were observed among different heavy metals in both pollen and nectar, indicating metal-specific translocation and partitioning into floral rewards.

### 2.2. Effects of Heavy Metal Exposure on Bumblebee Foraging Behavior

#### 2.2.1. Effects on Single-Flower Visit Duration

**Figure 3:**
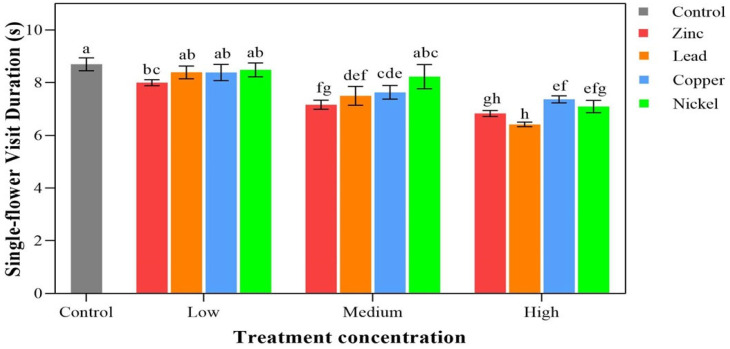
Effects of heavy metal addition to the soil on bumblebee single-flower visit duration. Values are presented as mean ± SE. Different letters within each metal indicate statistically significant differences among groups (*p* < 0.05).

Following heavy metal accumulation, the single-flower visit duration of bumblebees was significantly reduced compared with the control (8.70 ± 0.68 s). A clear concentration-dependent decrease in visit duration was observed across all treatments (Figure 3). Specifically, Zn exposure significantly reduced single-flower visit duration at all concentrations by 8.05–21.49% relative to the control (F_(2,6)_ = 60.857, *p = 0*.0001 < 0.001). Pb (F_(2,6)_ = 45.724, *p* = 0.006 < 0.01) exposure resulted in reductions ranging from 3.56% to 26.32%, while Cu (F_(2,6)_ = 45.724, *p = 0*.0002 < 0.001) caused decreases of 3.67–15.40%, exhibiting comparatively weaker effects similar to those observed under Pb treatment; however, statistically significant differences were detected only at medium and high concentrations. For Ni, single-flower visit duration declined at low and medium concentrations, but these reductions were not statistically significant. A significant decrease of 17.70% was observed only under high-concentration Ni treatment (F_(2,6)_ = 14.543, *p = 0*.005 < 0.01). At low concentration levels, although visitation duration was reduced relative to the control, no significant differences were detected among the four heavy metal treatments (*p* > 0.05). At medium concentrations, the Zn treatment resulted in the shortest visit duration (7.16 ± 0.17 S), with the magnitude of effects ranked as Zn > Pb > Cu > Ni. In contrast, at high concentrations, Pb exposure produced the shortest visitation duration (6.42 ± 0.08 S), and the severity of effects followed the order Pb > Zn > Ni > Cu.

#### 2.2.2. Effects on Flower Visitation Frequency

**Figure 4:**
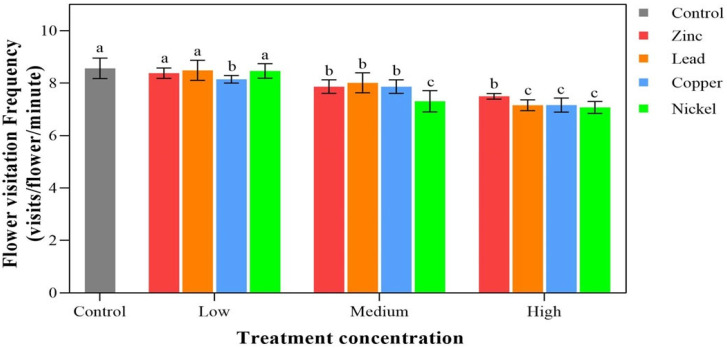
Effects of heavy metal addition to the soil on bumblebee flower visitation frequency. Values are presented as mean ± SE. Different letters within each metal indicate statistically significant differences among groups (*p* < 0.05).

Following heavy metal accumulation in blueberry flowers, bumblebee visitation frequency was significantly reduced at medium and high concentrations compared with the control (8.57 ± 0.39 flowers min^−1^) (Figure 4). Zn exposure decreased visitation frequency by 2.22–12.60%, with significant effects at medium and high concentrations (F_(2,6)_ = 14.937, *p* = 0.005 < 0.01). Pb (F_(2,6)_ = 12.240, *p* = 0.008 < 0.01) caused reductions of 0.93–16.57%, showing a similar trend. Cu (F_(2,6)_ = 14.384, *p* = 0.005 < 0.01) induced decreases of 5.02–16.45%, with significant effects at all concentrations (*p* < 0.05). Ni (F_(2,6)_ = 17.013, *p* = 0.003 < 0.01) reduced visitation rates by 1.28–17.50%, and significant effects at low concentrations were observed only under Cu treatment. At medium concentrations, Ni resulted in the lowest visitation frequency (7.31 ± 0.41 flowers min^−1^; Ni > Cu = Zn > Pb), while at high concentrations Ni, again, showed the strongest effect (7.07 ± 0.23 flowers min^−1^; Ni > Pb > Cu > Zn). Overall, heavy metal type and concentration significantly affected bumblebee flower visitation frequency and single-flower visit duration.

### 2.3. Effects of Heavy Metal Accumulation on Blueberry Reproductive Fitness

#### 2.3.1. Effects on Pollen Viability

**Figure 5:**
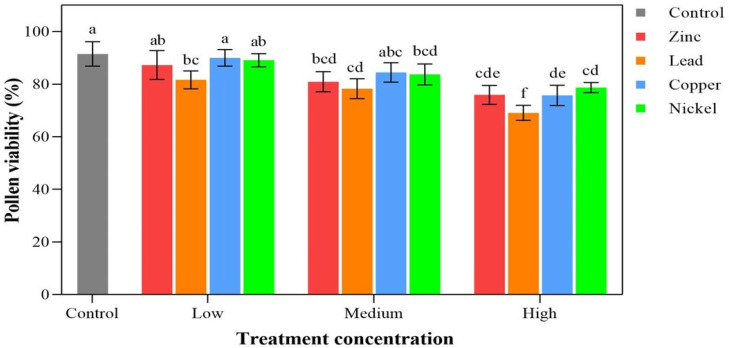
The effect of heavy metal addition to the soil on blueberry pollen viability. Values are presented as mean ± SE. Different letters within each metal indicate statistically significant differences among groups (*p* < 0.05).

Pollen viability declined significantly with increasing soil heavy metal concentrations, with pronounced reductions relative to the control, particularly at medium and high levels (Figure 5, *p* < 0.05). Compared with the control, pollen viability decreased by 4.61–17.03% under Zn (F_(2,12)_ = 8.511, *p* = 0.005 < 0.01) exposure, 10.81–24.48% under Pb (F_(2,12)_ = 18.507, *p* = 0.0002 < 0.001), 1.62–17.22% under Cu (F_(2,12)_ = 20.381, *p* = 0.0001 < 0.001), and 2.60–13.99% under Ni (F_(2,12)_ = 15.800, *p* = 0.0004 < 0.001), with all reductions being statistically significant (all *p* < 0.001).

At low concentrations, Pb exerted the strongest effect, with pollen viability reduced to 81.62 ± 3.37%, and the severity of impact ranked as Pb > Zn > Ni > Cu. Similar response patterns were observed at medium concentrations, although no significant differences were detected among the four heavy metals (*p* > 0.05). At high concentrations, Pb, again, showed the most pronounced effect, with pollen viability declining to 69.11 ± 2.87%, followed by Zn, Cu, and Ni (Pb > Zn > Cu > Ni). Overall, Pb consistently exerted the strongest inhibitory effect on pollen viability among the tested heavy metals.

#### 2.3.2. Effects on Pollen Deposition

**Figure 6:**
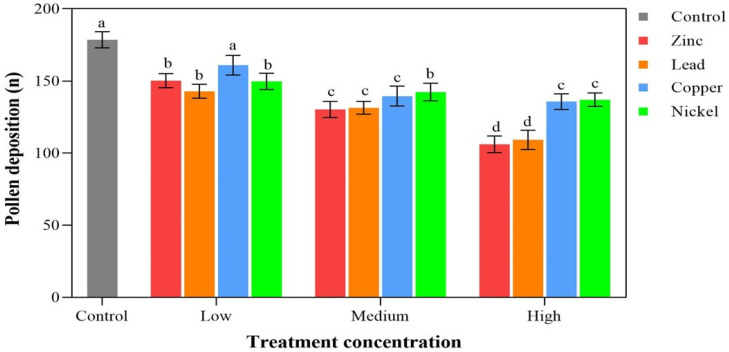
Treatment concentration effects of each metal on the number of pollen deposition. Values are presented as mean ± SE. Different letters within each metal indicate statistically significant differences among groups (*p* < 0.05).

Pollen deposition decreased significantly with increasing concentrations of all four heavy metals (Figure 6). Under different Zn (F_(2,27)_ = 164.546, *p* = 0.0000 < 0.001) treatments, pollen deposition declined by 15.90–40.59% relative to the control (178.60 ± 5.62), while Pb (F_(2,27)_ = 100.437, *p* = 0.0000 < 0.001) reduced deposition by 19.98–38.86%, Cu (F_(2,27)_ = 44.836, *p* = 0.0000 < 0.001) by 9.91–24.02%, and Ni (F_(2,27)_ = 13.368, *p* = 0.00009 < 0.001) by 16.18–23.29%, all showing significant effects (all *p* < 0.001).

At low concentrations, Pb had the strongest effect, with pollen deposition reduced to 142.90 ± 4.79, and the severity of impact ranked as Pb > Ni > Zn > Cu, although no significant differences were detected among Zn, Pb, and Ni (*p* > 0.05). At medium concentrations, Zn caused the largest reduction (130.20 ± 5.63), with the order of impact being Zn > Pb > Cu > Ni, and no significant differences were observed among Zn, Pb, and Cu (*p* > 0.05). At high concentrations, the trend was similar to that at medium concentrations, but pairwise differences among metals changed: Zn and Pb, as well as Cu and Ni, were not significantly different.

#### 2.3.3. Effect on Single-Fruit Weight

**Figure 7:**
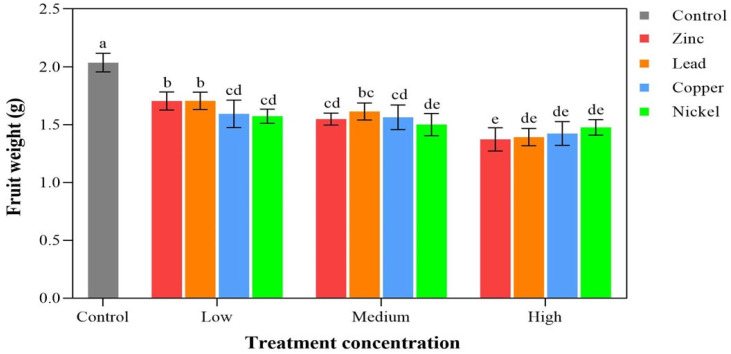
Effects of different heavy metal treatments on blueberry fruit weight. Values are presented as mean ± SE. Different letters within each metal indicate statistically significant differences among groups (*p* < 0.05).

Blueberry single-fruit weight decreased significantly with increasing heavy metal concentrations (Figure 7). Under Zn (F_(2,27)_ = 43.445, *p* = 0.0000 < 0.001) treatment, fruit weight declined by 16.25–32.51% relative to the control (2.03 ± 0.07 g), with significant differences observed. Pb (F_(2,27)_ = 47.269, *p =* 0.0000 < 0.001) showed similar effects, while Cu (F_(2,27)_ = 6.941, *p =* 0.004 < 0.01) reduced fruit weight by 21.67–30.04%, with no significant difference between low and medium concentrations (*p* > 0.05). Ni (F_(2,27)_ = 4.386, *p =* 0.022 < 0.05) caused a 22.66–27.58% decrease, with significant differences among concentrations (*p* < 0.05).

At low concentrations, Ni had the strongest effect (1.57 ± 0.06 g), with the order of impact being Ni > Cu > Pb = Zn; Zn and Pb, as well as Cu and Ni, did not differ significantly (*p* > 0.05). At medium concentrations, Ni, again, showed the largest reduction (1.50 ± 0.09 g), with impact severity ranked as Ni > Zn > Cu > Pb, and no significant differences among Zn, Pb, and Cu (*p* > 0.05). At high concentrations, differences among the four metals were not significant. Overall, heavy metal accumulation markedly reduced individual blueberry fruit weight.

## 3. Discussion

This study conducted a controlled experiment by adding aqueous solutions of heavy metal nitrates to the soil, aiming to investigate the accumulation patterns of four heavy metals (Zn, Pb, Cu, and Ni) in blueberry floral rewards, as well as their effects on the foraging behavior of the main pollinator, bumblebees, and on blueberry reproductive fitness.

### 3.1. Heavy Metal Accumulation Patterns in Blueberry Floral Rewards

This study demonstrates that the type and concentration of heavy metals strongly influence their accumulation patterns in blueberry floral rewards. While heavy metals accumulated in both pollen and nectar, pollen generally contained higher concentrations than nectar, consistent with observations in zucchini [18]. Accumulation patterns also varied among metals: Zn exhibited the highest enrichment in pollen, reflecting a strong accumulation capacity, similar to that reported for *Conyza canadensis* [19]. Cu showed no significant differences across low, medium, and high concentration treatments, indicating a tendency toward accumulation saturation, consistent with findings in the floral organs of *Pisum sativum* L. [20]. Ni accumulated significantly in pollen but displayed a lower overall accumulation capacity than Zn and Cu, whereas Pb showed the lowest concentrations, suggesting relatively weaker transport or enrichment into pollen, in agreement with observations on soil–pollen correlations for potentially toxic elements [21]. In nectar, heavy metal concentrations were generally slightly lower than in pollen. All four metals increased with rising soil concentrations, with Zn and Cu exhibiting relatively higher accumulation potential, reflecting general heavy metal accumulation trends in plants [22,23]. Notably, under the same soil concentration, differences in accumulation rates among metals were evident. In pollen, the difference between Zn and Cu widened with increasing concentration, reaching its maximum at high concentrations, whereas in nectar, no significant differences were observed between Zn and Cu across concentrations. Pb and Ni exhibited significant concentration-dependent variation in pollen, but not in nectar. Overall, heavy metal accumulation in both pollen and nectar displayed pronounced concentration-dependent effects, with marked variation among metal types.

### 3.2. Nectar-Mediated Heavy Metal Effects on Bumblebee Foraging

Our study revealed that heavy metal accumulation in blueberry floral rewards significantly affected the foraging behavior of pollinating bumblebees. Across all four heavy metal treatments, particularly at high concentrations, average single-flower visit duration decreased by over 20%, and visitation frequency declined with increasing concentration (Figure 3), potentially reducing pollination efficiency and ultimately affecting fruit quality. Previous studies support these findings: Meindl and Ashman reported that aluminum (Al) and nickel (Ni) in nectar significantly shortened bumblebee foraging time, likely because heavy metal accumulation alters nectar taste, enabling pollinators to detect metals and reduce visitation duration [7,24,25]. Similarly, Sivakoff and Gardiner found that bees spent significantly less time visiting sunflowers grown in Pb-contaminated soil, consistent with our results [26]. Furthermore, Meindl and Ashman demonstrated that Ni accumulation in the reproductive organs and floral rewards of *Streptanthus polygaloides* reduced visitation frequency by both bees and flies, aligning with our observations of Ni accumulation in blueberry pollen and nectar and its effect on bumblebee behavior [27,28]. High concentrations of heavy metals in floral resources diminish their attractiveness to pollinators and may also bioaccumulate through the food chain, negatively impacting pollinator health and reproduction. Some pollinators, including bees, can detect and avoid flowers contaminated with heavy metals [29]. Additionally, heavy metal exposure can impair insect nervous system function, reducing flight and foraging abilities, thereby decreasing visitation duration and frequency [30,31]. Heavy metals may also compromise sensory capabilities, limiting pollinators’ ability to locate and recognize flowers and, thus, reducing pollination efficiency [32]. Collectively, these findings provide strong evidence that heavy metal accumulation in floral rewards adversely affects pollinator visitation behavior, ultimately reducing plant reproductive success.

### 3.3. Effects of Heavy Metal Accumulation on Plant Reproductive Fitness

The effects of heavy metal accumulation on various aspects of plant reproductive fitness have been documented across multiple species. For example, Wani reported that soil Cd concentrations of 5.75 and 11.5 mg/kg reduced seed yield in *Cicer arietinum* by 14% and 19%, respectively, while also prolonging the flowering period [33]. Gür and Topdemir found that Cd, Cu, Hg, and Pb negatively affected both in vitro pollen germination and pollen tube growth in apricot and cherry, resulting in significantly lower fruit yields [34]. Similarly, Vogel-Mikuš showed that non-accumulator plants in heavy-metal-rich soils reduced seed production, impairing reproductive success [35]. Our previous work on zucchini demonstrated that soil addition of Pb and Ni decreased single-seed weight by approximately 10 mg. In the present study, all four heavy metal treatments, particularly at medium and high concentrations, led to elevated metal accumulation in blueberry floral rewards, which in turn altered bumblebee foraging behavior. As a result, pollen deposition on stigma tips decreased by approximately 20% relative to the control. Blueberry flowers are multi-ovule, averaging 106 ovules per flower, with at least half required for normal fruit development; insufficient fertilization impairs fruit formation [36]. Heavy-metal-induced reductions in pollen deposition, thus, led to incomplete ovule fertilization, resulting in a 20–30% decrease in average fruit weight compared with controls. Artificial pollination experiments indicate that heavy metal accumulation, per se, is not the primary cause of reduced fruit weight; rather, insufficient pollination due to decreased pollen deposition is the main factor [37,38]. Collectively, these findings suggest that insufficient pollination, mediated by heavy metal accumulation in floral rewards, ultimately limits yield by disrupting pollen transfer, fertilization, and seed and fruit development.

## 4. Materials and Methods

### 4.1. Experimental Materials

Blueberries (*Vaccinium* spp.) belong to the genus *Vaccinium* within the family Ericaceae and are evergreen or deciduous shrubs. Owing to their high anthocyanin content, blueberries are widely recognized as one of the top five health-promoting foods and have been extensively cultivated across China in recent years. Characterized by abundant flowering and the production of both nectar and pollen, blueberries represent an ideal experimental system for pollination-related studies, given their well-defined floral traits and suitability for controlled experimental manipulation. In the study region, blueberries have become an economically important crop. Blueberries typically flower annually in mid-May, with a flowering period of approximately two weeks. Each flower comprises five petals and five stamens. The cultivar used in this study, ‘Bluecrop’, produces approximately 21,000 pollen grains per flower, secretes 15–20 μL of nectar per flower, and contains about 100 ovules per flower [36,39]. As an entomophilous fruit crop, effective pollinators in the study region include *Apis mellifera ligustica*, *Bombus* spp., and *Apis cerana cerana*, among which bumblebees exhibit the highest pollination efficiency [36]. Accordingly, pollination behavior in this study was primarily assessed using the European bumblebee, a commercially available species widely employed for blueberry pollination in protected cultivation systems.

### 4.2. Experimental Design

Potted blueberry plants cultivated under protected conditions and exhibiting uniform growth were selected for the experiment. Each plant was grown individually in a plastic pot (32 × 33.5 cm) containing 25 L of nutrient soil, with one 5-year-old blueberry plant per pot. Within each treatment group, pots were arranged at 1 m intervals, and a distance of 2 m was maintained between different treatment groups. Four heavy metals commonly detected in the local environment (Zn, Pb, Cu, and Ni) were selected as experimental treatments. For each heavy metal, three concentration levels were established, with five replicate plants per concentration (n = 5). An additional 5 plants served as the control, resulting in a total of 65 experimental plants. Heavy metals were applied in aqueous solution form, prepared from analytical-grade nitrate salts: Zn(NO_3_)_2_·6H_2_O, Pb(NO_3_)_2_, Cu(NO_3_)_2_·6H_2_O, and Ni(NO_3_)_2_·6H_2_O (Liaoning Quanrui Reagent Co., Ltd., Jinzhou, China). The concentrations of heavy metal solutions were determined based on background levels measured in farmland soils from heavy-metal-polluted areas identified in our previous studies, which ranged from 212 to 527 mg·kg^−1^ for Zn, 141 to 372 mg·kg^−1^ for Pb, 109 to 374 mg·kg^−1^ for Cu, and 42 to 156 mg·kg^−1^ for Ni. The specific concentrations applied in this study are presented in Table 3.

Stock solutions of Zn, Pb, Cu, and Ni were prepared at a concentration of 1 mol·L^−1^. These stock solutions were diluted prior to each soil application. Each heavy metal was applied at three concentration gradients (low, medium, and high). The volumes of the 1 mol·L^−1^ stock solution added to each pot (after dilution) were as follows: Zn: 60 mL, 180 mL, 240 mL; Pb: 15 mL, 43.5 mL, 58 mL; Cu: 30 mL, 94 mL, 125 mL; Ni: 17 mL, 52 mL, 69 mL. To ensure heavy metal accumulation in floral organs, applications began one week prior to blueberry flowering. The diluted solutions were slowly and evenly poured into the soil along the edges of the pots using conical flasks (500 mL per application). Applications were performed at 2–3-day intervals until one week after flowering, for a total of five applications. Normal water and fertilizer management were resumed after flowering.

### 4.3. Flower Reward Collection and Detection

When approximately 30% or more of the flowers in the greenhouse experimental blueberry plants had opened successively after two weeks of heavy metal treatment, marking the onset of the flowering period, we collected blueberry flower rewards prior to releasing pollinating bumblebees. The experiment was conducted between 8:00 AM and 11:00 AM, which is the peak nectar secretion period for blueberry flowers. Nectar was collected using 0.4 mm glass capillary tubes, with a total of 80 flowers per treatment group. Subsequently, using tweezers, anthers from newly opened flowers were removed and placed into prepared Petri dishes lined with sulfur paper. Each treatment group collected anthers from 30 flowers, resulting in a total of 150 anthers. After returning to the laboratory, once the anthers were slightly dried, they were crushed using dissecting needles to remove impurities such as anther walls, yielding approximately 0.5 g of pollen. Nectar from all 80 capillary tubes was pooled into 5 mL centrifuge tubes, with approximately 1 mL collected per treatment. This nectar was then frozen for later use in heavy metal content analysis. Five plants were used per heavy metal treatment, ensuring that flowers from every plant were sampled. Both the collected pollen and nectar underwent freezing followed by microwave digestion. The heavy metal content was subsequently detected using ICP-MS (Thermo Scientific (Waltham, MA, USA)—iCAP Q). The inspection setup procedure is shown in Table 4.

### 4.4. Observation of Bumblebee Flower-Visiting Behavior

After collecting nectar and pollen samples for heavy metal content testing, we placed the experimental bumblebees, provided by the Bee Research Institute of Liaodong University (approximately 150 worker bees per hive), in a suitable location within the greenhouse one day prior to the experiment to allow for overnight acclimatization. The hive entrance was opened at 7:00 AM the following morning, at which point the bumblebees began to emerge from the hive to visit flowers. We recorded the bumblebees’ foraging behavior using a digital camera during three time periods: 8:00–9:00 AM, 11:00 AM–12:00 PM, and 2:00–3:00 PM. Each treatment group was filmed at least three times, primarily capturing the foraging behavior of individual bumblebees. This process was conducted consecutively over three sunny days. Subsequently, we processed the recorded video data of bumblebee foraging behavior using laboratory computer software. Timing commenced when the bumblebee landed on a blueberry flower and concluded when it departed, with the duration recorded as the single-flower visit duration. The number of flowers visited per unit time (1 min) was recorded as the foraging frequency.

### 4.5. Effects of Heavy Metal Accumulation on Blueberry Male–Female Fitness

#### 4.5.1. Effects of Heavy Metals on Pollen Viability

Pollen viability was assessed using the TTC method. During sampling, 10 flowers were collected with forceps and brought to the laboratory. Anthers were placed in sterile centrifuge tubes (1.5 mL), crushed, and impurities such as anther walls were removed. Then, 0.5% TCC phosphate buffer solution was added. The mixture was shaken to form a uniform suspension. Pollen viability was then observed and quantified under a microscope by calculating the percentage of red-stained pollen grains relative to the total pollen grains counted.

#### 4.5.2. Effect on Stigma Pollen Deposition

Stigma pollen deposition, defined as the number of pollen grains deposited on the stigma, was used as an indicator of pollination efficiency. For each treatment and the control, three newly opened inflorescences were randomly selected and tagged at the onset of anthesis. Flowers were then exposed to natural bumblebee visitation under greenhouse conditions. After three days, stigmas were collected from a minimum of 30 flowers per inflorescence using sterile tweezers and transferred to labeled centrifuge tubes for laboratory analysis. Pollen deposition was quantified using the Vaseline smear method. A thin, uniform layer of Vaseline was applied to a clean microscope slide, and each stigma was gently rolled across the slide to release pollen grains. Slides were examined under a light microscope (NOVEL XSZ-N107, ARI Group, Hefei, China) at 100× magnification, and the number of pollen grains deposited on each stigma was counted. The mean pollen load per stigma was calculated to assess pollination efficiency.

#### 4.5.3. Effect on Individual Fruit Weight

After the early-to-mid-stage blueberries in the experimental greenhouse had largely turned blue and reached maturity, we selected three fruit-bearing branches from each treatment group and the control group (after the entire inflorescence had set fruit). Each branch bore approximately 10–15 fruits of varying sizes. We then measured the average fruit weight for each treatment group to analyze how different levels of heavy metal accumulation affected individual fruit weight.

### 4.6. Data Analysis

Statistical analysis was performed using IBM SPSS Advanced Statistics 20.0 software. A two-way ANOVA was performed to assess the effects of heavy metal type and concentration on heavy metal levels in floral rewards (pollen and nectar). The independent variables were heavy metal type and treatment concentration, while the dependent variable was the heavy metal content. The independent variable was treatment concentration, while the dependent variable was the heavy metal content accumulated in floral rewards. For pollination behavior metrics (single-flower visit duration, visit frequency, pollen deposition, and single-fruit weight), one-way ANOVA was applied with treatment concentration as the independent variable. Multiple means were compared using the least significant difference (LSD) test at α = 0.05. GraphPad Prism 8.0.2 software was used for data visualization.

## 5. Conclusions

This study provides new insights into the effects of heavy metal accumulation on plant–pollinator mutualisms. We showed that heavy metals in soil can accumulate in the floral rewards of shrub crops such as blueberries, adversely affecting bumblebee foraging behavior. This results in insufficient pollen deposition, leading to pollen-limited ovule fertilization and reduced individual fruit weight, ultimately impairing blueberry reproductive fitness. Notably, this study is the first to simulate the natural pathway by which heavy metals affect both plants and pollinators: soil to floral rewards to pollinating bumblebees.

## Figures and Tables

**Table 1:** Analysis of variance (ANOVA) results: effects of different heavy metal types and concentrations on intermediate metal contents in pollen.

Source	df	MS	F	*p*
Metal Types	3	1163.440	190.347	<0.001
Concentration	3	779.069	127.461	<0.001
Types × Concentration	9	59.390	9.715	<0.001
Error	32	6.112		

**Table 2:** Analysis of variance (ANOVA) results: effects of different heavy metal types and concentrations on intermediate metal contents in nectar.

Source	df	MS	F	*p*
Metal Types	3	114.699	16.427	<0.001
Concentration	3	766.039	109.709	<0.001
Types × Concentration	9	16.789	2.404	0.032
Error	32	6.982		

**Table 3:** Heavy metal concentrations added to soil (mg·kg^−1^).

Metal	Concentration		
	Low	Medium	High
Zinc (Zn)	200	600	800
Lead (Pb)	150	450	600
Copper (Cu)	100	300	400
Nickel (Ni)	50	150	200

**Table 4:** ICP-MS parameters.

ICP-MS Parameter	Value
RF power	1550 W
Pump speed	40 rpm
S/C temperature	2.7 °C
Smpl depth	5 mm
Cool flow	14 L/min
Auxiliary flow	0.8 L/min
Nebulizer flow	1.122 L/min

## Data Availability

The data presented in this study are available on request from the corresponding author due to ongoing analyses and unpublished follow-up studies.
